# Report of Deaths in Buffalo for Dec. 1851

**Published:** 1852-02

**Authors:** W. L. G. Smith

**Affiliations:** City Clerk


*Report of Deaths in Buffalo, for Dec.* 1851.

BOARD OF HEALTH,
Buffalo, January, 1851.

Number of Deaths reported to the Board of Health in the month of December last.

Males, 37; Females, 23; Total, 60. Age—Adults, 29; Children, 31. Diseases—consumption, 6; convulsions, 4; lumbar abscess, 3; diarrhœa, 2; pneumonia, 4; typhoid fever, 7; dysentery, 2; unknown, 10; influenza, 2; anemia, 1; croup, 2; from burn, 1; malignant sore throat, 1; inflammation of brain, 1; do. of bowels, 2; peritonitis, 1; hydrocephalus, 1; apoplexy, 1; old age, 1; still born, 2; intemperance, 2; small pox, 1; casualty, 1; painter's colic, 1; frozen to death, 1.

By order of the Board,

W. L. G. SMITH, City Clerk.

